# Topological Quantum Codes from Lattices Partition on the *n*-Dimensional Flat Tori

**DOI:** 10.3390/e23080959

**Published:** 2021-07-27

**Authors:** Edson Donizete de Carvalho, Waldir Silva Soares, Eduardo Brandani da Silva

**Affiliations:** 1 Department of Mathematics, UNESP, Ilha Solteira, SP 15385-000, Brazil; edson.donizete@unesp.br; 2 Departament of Mathematics, UEM, Av. Colombo 5790, Maringa, PR 87020-900, Brazil; 3 Department of Mathematics, UTFPR, Pato Branco, PR 85503-390, Brazil; waldirjunior@utfpr.edu.br

**Keywords:** color codes, surface codes, toric codes, flat torus, lattice partition, 81P70, 52C07, 81Q35

## Abstract

In this work, we show that an *n*-dimensional sublattice $\Lambda^{\prime }=m\Lambda$ of an *n*-dimensional lattice Λ induces a $G=Z_{m}^{n}$ tessellation in the flat torus $T_{\beta^{\prime }}=R^{n}/\Lambda^{\prime }$, where the group *G* is isomorphic to the lattice partition $\Lambda /\Lambda^{\prime }$. As a consequence, we obtain, via this technique, toric codes of parameters $\left[\left[2m^{2},2,m\right]\right]$, $\left[\left[3m^{3},3,m\right]\right]$ and $\left[\left[6m^{4},6,m^{2}\right]\right]$ from the lattices $Z^{2}$, $Z^{3}$ and $Z^{4}$, respectively. In particular, for n=2, if $\Lambda_{1}$ is either the lattice $Z^{2}$ or a hexagonal lattice, through lattice partition, we obtain two equivalent ways to cover the fundamental cell $P_{0}^{\prime }$ of each hexagonal sublattice $\Lambda^{\prime }$ of hexagonal lattices Λ, using either the fundamental cell $P_{0}$ or the Voronoi cell $V_{0}$. These partitions allow us to present new classes of toric codes with parameters $\left[\left[3m^{2},2,m\right]\right]$ and color codes with parameters $\left[\left[18m^{2},4,4m\right]\right]$ in the flat torus from families of hexagonal lattices in $R^{2}$.

## 1. Introduction

The concepts and ideas of classical error-correcting codes theories were an inspiration and model to build quantum error-correcting codes. In 1996, a fundamental step in the quantum coding theory was made due to the discovery of a new class of codes, now known as CSS codes, by Robert Calderbank, Peter Shor and Andrew Steane [1,2] and which originated a richer code structure—the stabilizer quantum codes [3].

Superposition of states is a fundamental property for the processing of quantum information. These superpositions are very fragile, and they can be destroyed by interactions of the quantum system with the surrounding environment. Local stabilizer codes offer an alternative solution to this problem.

Kitaev [4] proposed a particular class of stabilizer codes, which is associated with a $Z^{2}$ lattice. These codes depend on the topology of a surface, and they belong to the general class of topological quantum codes (TQC), which, in turn, belong to the class of stabilizer quantum codes. In these codes, we encode quantum words in non-local degrees of freedom of strongly correlated quantum systems that have topological order, such as certain gauge theories in a lattice, or in condensed matter systems. Due to this non-local encoding, these quantum words are intrinsically resistant to the disturbing effects of noise, as long as this noise is local in the sense of not affecting the global (topological) properties of the system. This construction is quite remarkable because it is based on an intrinsically physical mechanism that makes the topological system capable of self-correcting local errors by itself. This means that, in a topological code, we do not need to detect and repair quantum errors from outside the system as in usual non-topological codes. It is the physical properties of a system that produce a mechanism for protecting the encoded quantum states. This mechanism is controlled by interactions described by a Hamiltonian in certain lattices or lattices immersed in surfaces with non-trivial topology. The ground state of these Hamiltonians exhibits topological order, one of the signatures being the existence of a type of ground state degeneration that is robust to local disturbances. This is due to the existence of an energy gap that separates the ground state from the rest of the excited states in the Hamiltonian spectrum. Furthermore, this degeneration depends on the network topology where the strongly correlated system Hamiltonian is defined. Due to this topological order, these states have very relevant entanglement properties. Intuitively, a topological order is a certain kind of long-range entanglement in the ground and excited states of a given quantum system. The topology may provide further protection in ordinary circuit quantum computing. For instance, the combination of topological distribution and dynamical decoupling will further strengthen the error correction capability [5,6]. The simulation of topological properties in circuit quantum computing system has also been attracting attention recently [7].

In his work, Kitaev showed that the construction procedure of stabilizing codes can also be generated from the tessellation of a three-dimensional flat torus by cubes and from the tessellation of the four-dimensional flat torus by hypercubes. To build TQC, we may consider a tiling (tessellation) of a surface, or a hypersurface for dimensions n≥3. The most important classes of TQC are the surfaces codes and the color codes. The surface codes obtained from the flat tori are called toric codes. In the two-dimensional flat torus, the stabilizer operators are attached to the vertices and faces of the polygons, which tessellate the torus, and the encoded qubits are related to the homological non-trivial cycles on the torus surface. In the toric codes obtained from three-dimensional flat torus, the stabilizer operators *X* and *Z* are attached to the cells and edges which tessellate the torus surface, and the encoded qubits are related to the homological non-trivial faces on the torus. In a similar way to the toric codes obtained from four-dimensional flat torus, the stabilizer operators *X* and *Z* are attached to the cells and edges of the paralellepiped, which tessellates the torus surface, and the encoded qubits are related to the homological non-trivial faces on each two-dimensional flat torus.

Color codes, introduced by Bombin and Martin-Delgado [8], are built on three-valent tessellations, which have three-colorable faces, that is, it is possible to color each face using three different colors such that the neighbor faces have different colors. There are two stabilizer operators attached in each face of the tessellation. This leads to encoding twice as many qubits than surface codes as we can see in [9]. Color codes obtained from two-dimensional flat torus were proposed on hexagon lattices [10], i.e., lattices in which the Voronoi cells are given by regular hexagons (three-valent tessellations), and where the qubits are attached to each vertex of the regular hexagons that tessellate the torus. These two-dimensional lattices are known in quantum coding theory by honeycomb lattices (because of the shape of its Voronoi cells). We need to determine the possible regular hexagons that tessellate the torus, and analyze the paths on the hexagonal tessellation to obtain the shortest cycle for each color of the three-colored faces of the torus.

In the current work, an alternative approach is considered. We propose an algebraic and geometric characterization for the construction of topological quantum codes from a family of flat tori since we may characterize topological codes on the flat torus as cosets of lattice quotients, putting them in the context of lattice theory. These facts have not yet been exploited in quantum coding theory.

The lattice theory is rich in algebraic and geometric properties. Among the geometric properties, we are interested in lattice partitions, with emphasis on the parallelepiped and Voronoi partitions—different and equivalent ways of covering the space $R^{n}$.

Associated with a sublattice $\Lambda^{\prime }$ of an *n*-dimensional lattice Λ, there is a fundamental parallelepiped $P_{0}^{\prime }$ of the partition of lattice $P^{\prime }$ associated to the sublattice $\Lambda^{\prime }$. Similarly, there is a fundamental parallelepiped $P_{0}$ of the lattice partition P associated with the sublattice Λ. The lattice partition $\Lambda /\Lambda^{\prime }$ is an additive group of cardinality *l*, and from the geometric point of view, it guarantees that the fundamental region $P_{0}^{\prime }$ is covered with *l* fundamental regions $P_{0}$. From the bases $\beta =\{u_{1},\ldots ,u_{n}\}$ and $\beta^{\prime }=\left\{mu_{1},\ldots ,mu_{n}\right\}$ associated with the lattices Λ and $\Lambda^{\prime }$, respectively, we obtain the flat torus $T_{\beta^{\prime }}=R^{n}/\Lambda^{\prime }$, geometrically obtained by identification of opposite faces (parallel to the basis vectors $\beta^{\prime }$) of the fundamental region $P_{0}^{\prime }$.

As a consequence of this algebraic/geometrical characterization of $T=R^{n}/\Lambda^{\prime }$, for n=2, we reproduce the toric codes proposed by Kitaev [4] with parameters $\left[\left[2m^{2},2,m\right]\right]$. For n=3, we obtain the toric codes with parameters $\left[\left[3m^{3},3,m\right]\right]$, see [11], and for n=4, we obtain the toric codes proposed by Breuckman et al. [12] with parameters $\left[\left[6m^{4},6,m^{2}\right]\right]$.

If Λ is a hexagonal lattice, we prove that the $Z_{m}^{2}$-tessellation of each flat torus $T_{\beta^{\prime }}$ is covered by $m^{2}$ regular hexagons derived from regular tessellation {6,3} in $R^{2}$, and also covered by $2m^{2}$ regular triangles derived from the dual tessellation {3,6}. As a consequence, we present a new class of toric codes of parameters $\left[\left[3m^{2},2,m\right]\right]$ obtained from hexagonal lattices (honeycomb lattices). These lattices were first considered by Kitaev in [13]. The honeycomb lattice is very important since it is a topologically ordered system involving only two-body interactions [14] and have been also used to build new quantum memories [15].

Finally, considering sublattices $\Lambda^{\prime \prime }$ with basis $\beta^{\prime \prime }=\left\{3mu_{1},3mu_{2}\right\}$ and index $9m^{2}$ in the hexagonal lattice Λ, with basis $\beta_{1}=\left\{u_{1},u_{2}\right\}$, we prove that Λ induces a $Z_{3m}^{2}$-tessellation on each flat torus $T_{\beta^{\prime \prime }}$, and it is covered by $9m^{2}$ regular hexagons derived from regular tessellation {6,3} in $R^{2}$. As a consequence of this algebraic/geometrical characterization of $T=R^{2}/\Lambda^{\prime \prime }$, we systematize the process of generating classes of color codes with parameters $\left[\left[18m^{2},4,4m\right]\right]$.

## 2. Basic Results

In this section, we present basic concepts and results of lattice theory and tessellations of the flat torus, which are used for the development of the current work.

### 2.1. Lattices in $R^{n}$

Roughly speaking, a lattice is a set of points of $R^{n}$, which is isomorphic to the additive group $Z^{n}$. This algebraic structure leads us to the study of subgroups (sublattices) and partitions (coset decompositions) induced by such subgroups. Geometrically, a lattice is endowed with the properties of the space in which it is embedded, such as the Euclidean metric and the notion of volume in $R^{n}$.

Formally, a lattice Λ is a set of points given by $\Lambda =\{x\in R^{n}:x=\sum_{i=1}^{m}\lambda_{i}u_{i}\;\mathrm{and}\;\lambda_{i}\in Z\}$, where $\left\{u_{1},u_{2},\ldots ,u_{m}\right\}$ is a set of linearly independent vectors in $R^{n}$, and it is called the lattice basis. We say that Λ is a lattice with rank *m* in $R^{n}$. If m=n, we say the lattice has full rank in $R^{n}$. In the current work, we are considering only full rank lattices.

If Λ is a *n*-dimensional lattice with basis $\left\{u_{1},u_{2},\ldots ,u_{n}\right\}$, its generator matrix is given by the following:

$$M=\left(\begin{array}{cccc} u_{11} & u_{12} & \cdots & u_{1n}\\ u_{21} & u_{22} & \cdots & u_{2n}\\ \vdots & \vdots & \ddots & \vdots \\ u_{n1} & u_{n2} & \cdots & u_{nn} \end{array}\right),$$

where $u_{i}=\left(u_{i1},u_{i2},\ldots ,u_{in}\right)$, for i=1,2,…,n. A lattice has full rank if its generator matrix has full rank. The matrix $G=MM^{T}$ is called the Gram matrix of the lattice Λ, where $M^{T}$ denotes the transpose of *M*. We can also define $\Lambda =\{x=\lambda M\;|\;\lambda \in Z^{n}\}$. The determinant of the lattice Λ is defined as the determinant of the matrix *G*, that is, Det(Λ)=Det(G).

The following examples of lattices of full rank in $R^{2}$ are important in what follows.

**Example** **1.** *Let $\left\{u_{1},u_{2}\right\}$ be a basis in $R^{2}$.*

*1.*  *If $u_{1}=\left(1,0\right)$ and $u_{2}=\left(0,1\right)$, we obtain the lattice $Z^{2}$, whose associated generator matrix is given by the following:*
  $$M=\left(\begin{array}{cc} 1 & 0\\ 0 & 1 \end{array}\right).$$
*2.*  *If $u_{1}=\left(1,0\right)$ and $u_{2}=\left(-\frac{1}{2},\frac{\sqrt{3}}{2}\right)$, we obtain the hexagonal lattice $A_{2}$, whose generator matrix is given by the following:*
  $$M=\left(\begin{array}{cc} 1 & 0\\ -\frac{1}{2} & \frac{\sqrt{3}}{2} \end{array}\right).$$

**Example** **2.**

*1.*  *Let $\left\{u_{1},u_{2},u_{3}\right\}$ be a basis in $R^{3}$. If $u_{1}=\left(1,0,0\right),\;u_{2}=\left(0,1,0\right)$ and $u_{3}=\left(0,0,1\right)$, we obtain the lattice $Z^{3}$, whose associated generator matrix is given by the following:*
  $$M=\left(\begin{array}{ccc} 1 & 0 & 0\\ 0 & 1 & 0\\ 0 & 0 & 1 \end{array}\right).$$
*2.*  *Let $\left\{u_{1},u_{2},u_{3},u_{4}\right\}$ be a basis in $R^{4}$. If $u_{1}=\left(1,0,0,0\right),\;\;u_{2}=\left(0,1,0,0\right),\;\;u_{3}=\left(0,0,1,0\right)$ and $u_{4}=\left(0,0,0,1\right)$, we obtain the lattice $Z^{4}$, whose associated generator matrix is given by the following:*
  $$M=\left(\begin{array}{cccc} 1 & 0 & 0 & 0\\ 0 & 1 & 0 & 0\\ 0 & 0 & 1 & 0\\ 0 & 0 & 0 & 1 \end{array}\right).$$

Given a lattice Λ, a subset $\Lambda^{\prime }\subset \Lambda$ is a sublattice if $\Lambda^{\prime }$ itself is a lattice, i.e., $\Lambda^{\prime }$ is an additive subgroup of Λ.

The sublattice $\Lambda^{\prime }$ induces a partition of Λ into lateral classes. The set of these lateral classes is a quotient lattice, denoted by $\Lambda /\Lambda^{\prime }$, with cardinality given by the following Equation (1):

(1) $$l=|\Lambda /\Lambda^{\prime }|.$$

In this case, we say that $\Lambda^{\prime }$ has index *l* in Λ. The sublattice $\Lambda^{\prime }$ can also be characterized as $\Lambda^{\prime }=\left\{x=\lambda BM\;|\;\lambda \in Z^{n}\right\}$, where *M* is the generator matrix associated of the lattice Λ, and *B* is a square matrix of integers, whose absolute value of determinant is greater than 1. The index of $\Lambda^{\prime }$ in Λ can also be characterized by $|\Lambda /\Lambda^{\prime }\left|=|det\left(B\right)\right|$.

Remark 1 provides a matrix description of how to obtain families of sublattices $\Lambda^{\prime }$ from a lattice Λ of index $l=m^{n}$.

**Remark** **1.** *From any lattice* Λ *in $R^{n}$ with basis $\beta =\{u_{1},u_{2},\ldots ,u_{n}\}$ and generating matrix M, we can obtain a family of sublattices $\Lambda^{\prime }$ in* Λ *with index $m^{n}$, generated by the integer basis $\beta_{n}=\left\{mu_{1},mu_{2},\ldots ,mu_{n}\right\}$ and generating matrix $M^{\prime }=B^{\prime }M$, with the following:*

$$B^{\prime }=\left(\begin{array}{cccccc} m & 0 & 0 & \ldots & 0 & 0\\ 0 & m & 0 & \ldots & 0 & 0\\ \vdots & \vdots & \vdots & \vdots & \vdots & \vdots \\ 0 & 0 & 0 & \ldots & 0 & m \end{array}\right).$$

**Example** **3.**

*1.*  *For the lattice $Z^{2}$ with basis $\beta =\{u_{1},u_{2}\}$ and generating matrix M given by item (1) of Example 1, we can obtain a family of sublattices $\Lambda^{\prime }=mZ^{2}$ in $Z^{2}$ with index $m^{2}$. Just take $\beta^{\prime }=\left\{mu_{1},mu_{2}\right\}$ as a basis and $M^{\prime }=B^{\prime }M$ as the generating matrix for each sublattice $\Lambda^{\prime }=mZ^{2}$ in $Z^{2}$, where $B^{\prime }$ is the matrix given by Remark 1 of rank 2. Thus, each sublattice $\Lambda^{\prime }=mZ^{2}$ in $Z^{2}$ has index $m^{2}$ since $det\left(B^{\prime }\right)=m^{2}$.*
*2.*  *For the lattice $Z^{3}$ with basis $\beta =\{u_{1},u_{2},u_{3}\}$ and generating matrix M given by item (1) of Example 2, we can obtain a family of sublattices $\Lambda^{\prime }=mZ^{3}$ in $Z^{3}$ with index $m^{3}$. Simply take $\beta^{\prime }=\left\{mu_{1},mu_{2},mu_{3}\right\}$ as a basis and $M^{\prime }=B^{\prime }M$ as the generating matrix for each sublattice $\Lambda^{\prime }=mZ^{3}$ in $Z^{3}$, where $B^{\prime }$ is the matrix given by Remark 1 of rank 3. Thus, each sublattice $\Lambda^{\prime }=mZ^{3}$ in $Z^{3}$ has index $m^{3}$ since $det\left(B^{\prime }\right)=m^{3}$.*
*3.*  *For the lattice $Z^{4}$ with basis $\beta =\{u_{1},u_{2},u_{3},u_{4}\}$ and generating matrix M given by item (2) of Example 2, we can obtain a family of sublattices $\Lambda^{\prime }=mZ^{4}$ in $Z^{4}$ with index $m^{4}$. Simply take $\beta^{\prime }=\left\{mu_{1},mu_{2},mu_{3},mu_{4}\right\}$ as a basis and $M^{\prime }=B^{\prime }M$ as the generating matrix for each sublattice $\Lambda^{\prime }=mZ^{4}$ in $Z^{4}$, where $B^{\prime }$ is the matrix given by Remark 1 of rank 4. Thus, each sublattice $\Lambda^{\prime }=mZ^{4}$ in $Z^{4}$ has index $m^{4}$, since $det\left(B^{\prime }\right)=m^{4}$.*

**Example** **4.**

*1.*  *Let* Λ *be a hexagonal sublattice of $A_{2}$ with basis $\beta =\{u_{1},u_{2}\}$; generating matrix M, we can obtain a family of sublattices $\Lambda^{\prime }$ in* Λ *with index $m^{2}$. We take $\beta^{\prime }=\left\{mu_{1},mu_{2}\right\}$ as the basis, generating matrix $M^{\prime }=B^{\prime }M$ for each sublattice $\Lambda^{\prime }$, where $B^{\prime }$ is the matrix given in Remark 1, of rank 2. Thus, each sublattice $\Lambda^{\prime }$ in* Λ *has index $m^{2}$ since $det\left(B^{\prime }\right)=m^{2}$.*
*2.*  *Let* Λ *be a hexagonal sublattice of $A_{2}$ with basis $\beta =\{u_{1},u_{2}\}$, generating matrix M. We can obtain a family of sublattices $\Lambda^{\prime \prime }$ in* Λ *with index $9m^{2}$, generated by the basis $\beta^{\prime \prime }=\left\{3mu_{1},3mu_{2}\right\}$ and generating matrix $M^{\prime \prime }=B^{\prime \prime }M$, with the following:*
  $$B^{\prime \prime }=\left(\begin{array}{cc} 3m & 0\\ 0 & 3m \end{array}\right).$$

Let $\left\{u_{1},\ldots ,u_{n}\right\}$ be a basis of an *n*-dimensional lattice Λ. The fundamental parallelepiped of Λ is the set of all points in $R^{n}$, which are convex linear combination of vectors of the basis, with coefficients between zero and one, that is, the following:

(2) $$P_{0}=\left\{x=\sum_{i=1}^{n}\alpha_{i}u_{i};\;0\leq \alpha_{1},\ldots ,\alpha_{n}<1\right\}\;.$$

Let $\Lambda^{\prime }$ be a sublattice of Λ, whose generator matrices are given by *M* and $M^{\prime }=BM$, respectively. It can be proved that the following holds:

(3) $$|\Lambda /\Lambda^{\prime }|=\frac{volume(P_{0}^{\prime })}{volume(P_{0})}=\left|det\left(B\right)\right|,$$

where $volume(P_{0}^{\prime })$ and $volume(P_{0})$ denote the volume of the fundamental parallelepiped $P_{0}^{\prime }$ (associated to the sublattice $\Lambda^{\prime }$) and the volume of the parallelepiped $P_{0}$ (associated to the lattice Λ), respectively. Equation (3) shows that the fundamental parallelepiped of the lattice Λ induces a partition in the fundamental parallelepiped of the $\Lambda^{\prime }$ sublattice, that is, a covering of the fundamental parallelepiped of $\Lambda^{\prime }$ by *l* copies of the fundamental parallelepiped of Λ, where *l* denotes the index of the sublattice $\Lambda^{\prime }$ in Λ, given by Equation (1).

In this direction, in the next section, we will characterize the coverings of $R^{n}$ via a parallelepiped partition induced by lattices.

### 2.2. Lattice Partitions

We say that a collection of sets $\left\{S_{i}\right\}$ covers the Euclidean space $R^{n}$ if every point of $R^{n}$ is in one of the sets of the collection $\left\{S_{i}\right\}$, that is, $\cup_{i}S_{i}=R^{n}$. A collection of sets $\left\{S_{i}\right\}$ that covers the space such that $S_{i}^{\circ }\cap S_{j}^{\circ }=\emptyset$ for all i≠j is called a partition of $R^{n}$, where $S_{i}^{\circ }$ denotes the interior of $S_{i}$, considering the Euclidean norm. In this work, we are interested in parallelepiped partitions and Voronoi partitions obtained from *n*-dimensional lattices Λ in $R^{n}$.

**Definition** **1.** *A fundamental cell $P_{0}$ of a lattice* Λ *is a bounded set such that, when translated by points of the lattice* Λ*, it generates a partition $P=\{P_{\lambda }:\lambda \in \Lambda \}$ from $R^{n}$. Thus, the following are true.*

*1.*  *Each cell $P_{\lambda }$ is obtained by translating $P_{0}$ by a lattice point λ:*
  $$P_{\lambda }=P_{0}+\lambda =\left\{x:\left(x-\lambda \right)\in P_{0}\right\}\;.$$
*2.*  *The cells do not intersect, that is, ${P_{\lambda }}^{\circ }\cap {P_{\lambda^{\prime }}}^{\circ }\neq \emptyset$ for all $\lambda \neq \lambda^{\prime }\in \Lambda$, where $A^{\circ }$ denotes the interior of a set $A\subset R^{n}$.*
*3.*  *the union of all cells covers the whole space $R^{n}$, i.e., $\cup_{\lambda \in \Lambda }P_{\lambda }=R^{n}$.*

An important geometric property is that all cells belonging to the lattice partition $\left\{P_{\lambda }\right\}$ are congruent.

From (1) of Definition 1, each point $x\in R^{n}$ can be written uniquely as follows:

(4) $$x=\lambda +x_{e}\;\;where\;\;\lambda \in \Lambda \;\;and\;\;x_{e}\in P_{0}.$$

Each point λ∈Λ is an approximation of $x\in R^{n}$ satisfying (4). The points $x_{e}\in P_{0}$ satisfying (4) may be seen as the error in the approximation of each point $x\in R^{n}$ by a lattice point λ∈Λ. We denote this approximation by $\lambda =Q_{\Lambda }\left(x\right)$. We call $Q_{\Lambda }\left(x\right)$ the quantization of *x*.

The other very important partition of the space is the Voronoi partition, which uses a nearest-neighbor rule. Let ∥.∥ be the Euclidean norm on $R^{n}$. The distance of a point *x* in $R^{n}$ from Λ is defined as the following:

$$∥x-\Lambda ∥=\min_{\lambda \in \Lambda }∥x-\lambda ∥\;.$$

The nearest-neighbor quantizer $Q_{\Lambda }^{N}$ maps *x* to its closest lattice point:

$$Q_{\Lambda }^{N}\left(x\right)=\arg\min_{\lambda \in \Lambda }∥x-\lambda ∥\;.$$

Then, we define the following.

**Definition** **2.** *The Voronoi cell associated to an n-dimensional lattice point λ∈Λ is the set of all points, which are quantized to λ, i.e., $V_{\lambda }=\left\{x\in R^{n}:Q_{\Lambda }^{N}\left(x\right)=\lambda \right\}$.*

From the definition of $Q_{\Lambda }^{N}$, we have that the resulting Voronoi cells are congruent. For each lattice Λ, we denote by $V_{0}$ the Voronoi cell associated to the lattice point 0∈Λ. When we translate $V_{0}$ by lattice points λ∈Λ, we obtain a partition $V=\{V_{\lambda }:\lambda \in \Lambda \}$ of $R^{n}$, whose union of all cells covers the whole space $R^{n}$, where $V_{\lambda }=V_{0}+\lambda =\left\{x\in R^{n}:\left(x-\lambda \right)\in V_{0}\right\}$. We also have that all Voroni regions belonging to the Voronoi partition $\left\{V_{\lambda },\lambda \in \Lambda \right\}$ are congruent. We have that the Voronoi partition generated by the nearest-neighbor quantizer $Q_{\Lambda }^{N}$ satisfies the properties in Definition 1. However, the simplest lattice partition is the parallelepiped partition generated by some lattice basis $\left\{u_{1},\ldots ,u_{n}\right\}$. In what follows, $P_{0}$ denotes the fundamental parallelepiped given by (2).

**Remark** **2.** *It should be noted that the fundamental cell $P_{0}$ depends on the choice of the basis vectors of the lattice* Λ*. However, the fundamental cell $P_{\lambda }$ of the partition $P=\{P_{\lambda }:\lambda \in \Lambda \}$ is obtained by the translation of the fundamental parallelepiped $P_{0}$ by the lattice point λ. The Voronoi cell $V_{\lambda }$ of partition $V=\{V_{\lambda }:\lambda \in \Lambda \}$ depends on the point λ∈Λ, but it can also be obtained by the translation of the Voronoi cell $V_{0}$ by the lattice point λ, regardless of the choice of vectors of the base of the lattice* Λ*.*

**Remark** **3.**

*1.*  *The fundamental parallelepiped $P_{0}$ and the fundamental Voronoi region $V_{0}$ associated with the lattice point $\lambda_{0}=\left(0,0\right)\in Z^{2}$ are given by squares, and they are the same apart a translation, that is, the partitions $P=\{P_{\lambda }:\lambda \in Z^{2}\}$ and $V=\{V_{\lambda }:\lambda \in Z^{2}\}$ obtained from the lattice $Z^{2}$ are congruent.*
*2.*  *The fundamental parallelepiped $P_{0}$ and the fundamental Voronoi region $V_{0}$ associated with the lattice point $\lambda_{0}=\left(0,0,0\right)\in Z^{3}$ are given by cubes, and they are the same apart a translation, that is, the partitions $P=\{P_{\lambda }:\lambda \in Z^{3}\}$ and $V=\{V_{\lambda }:\lambda \in Z^{3}\}$ obtained from the lattice $Z^{3}$ are congruent.*
*3.*  *The fundamental parallelepiped $P_{0}$ and the fundamental Voronoi region $V_{0}$ associated with the lattice point $\lambda_{0}=\left(0,0,0,0\right)\in Z^{4}$ are given by hypercubes, and are the same apart a translation, that is, the partitions $P=\{P_{\lambda }:\lambda \in Z^{4}\}$ and $V=\{V_{\lambda }:\lambda \in Z^{4}\}$ obtained from the lattice $Z^{4}$ are congruent.*

By Remark 3, we conclude that lattices in $Z^{2}$, $Z^{3}$ and $Z^{4}$ reproduce parallelepiped partitions P and Voronoi partitions V that are congruent (equivalent).

**Figure 1:**
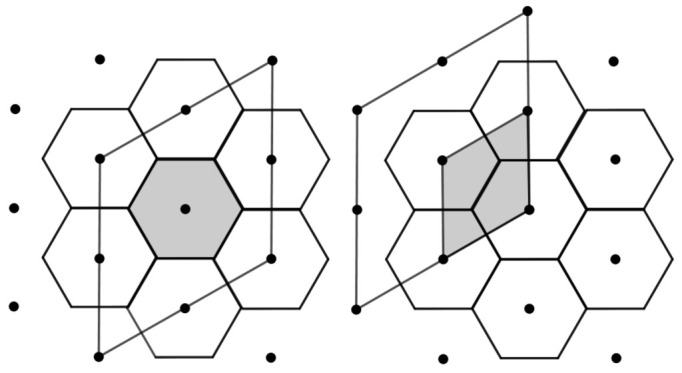
Voronoi and fundamental regions associated to the hexagonal lattice Λ.

**Example** **5.** *Let* Λ *be the hexagonal lattice given in Example 4. Figure 1 shows on the left the fundamental Voronoi cell $V_{0}$ of $V=\{V_{\lambda }:\lambda \in \Lambda \}$, and on the right, we have the fundamental parallelepiped cell $P_{0}$ of $P=\{P_{\lambda }:\lambda \in \Lambda \}$.*

*The fundamental cell $P_{0}$ is a parallelogram with edges parallel to the vectors $u_{1}$ and $u_{2}$ of the basis β, given in Example 4. On the other hand, the fundamental cell $V_{0}$ (Voronoi cell) is a regular hexagon.*

As we see from Figure 1, for a hexagonal lattice Λ, the fundamental parallelepiped $P_{0}$ given by a parallelogram and the Voronoi region $V_{0}$ given by a regular hexagon associated with any lattice point λ∈Λ are not equal apart translations. However, we will see below that the partitions $P=\{P_{\lambda }:\lambda \in \Lambda \}$ and $V=\{V_{\lambda }:\lambda \in \Lambda \}$ obtained by the lattice Λ are congruent (equivalent). For this, we will consider, below, Definition 3, Lemma 1 and the Proposition 1, which will be of fundamental importance for the construction of new surface and color codes in the flat torus from hexagonal lattices Λ.

**Definition** **3.** *For a given lattice partition P with a fundamental cell $P_{0}$, the modulo fundamental cell operation is defined as the following:*

(5) $$x\;\;mod\;\;P_{0}=x_{e}=x-Q_{\Lambda }\left(x\right),$$

*where $Q_{\Lambda }\left(x\right)$ and $x_{e}$ are the quantization and quantization error, respectively, induced by the partition P. We call this a modulo–lattice operation, and we use the notation xmodΛ.*

**Lemma** **1** ([16]). *The result of the modulo–lattice operation is the same for any fundamental cell of the lattice, up to a shift by a lattice point:*

(6) $$\left(x\;\;mod\;\;P_{0}\right)-\left(x\;\;mod\;\;V_{0}\right)\in \Lambda ,$$

*where $P_{0}$ and $V_{0}$ are the fundamental parallelepiped cell and the Voronoi cell of* Λ*, respectively. We also have that all fundamental cells $P_{0}$ and $V_{0}$ of* Λ *are equal up to a modulo–lattice operation:*

(7) $$V_{0}\;\;mod\;\;P_{0}=P_{0}.$$

We have in Example 1 two different lattices partitions of the lattice $\Lambda_{1}$, given by $V=\{V_{\lambda }:\lambda \in \Lambda_{1}\}$ and $P=\{P_{\lambda }:\lambda \in \Lambda_{1}\}$. From Lemma 1, we have that $V_{0}\;\;mod\;\;P_{0}=P_{0}$.

In a similar way, from Examples 3 and 4, we also have two different lattice partitions of the lattice $\Lambda_{1}$, given by $V=\{V_{\lambda }:\lambda \in \Lambda_{1}\}$ and $P=\{P_{\lambda }:\lambda \in \Lambda_{1}\}$. From Lemma 1, we have that $V_{0}\;\;mod\;\;P_{0}=P_{0}$.

From the next proposition, we have that these two partitions, V and P, are equivalent.

**Proposition** **1** ([16]). *For the partitions P and V with fundamental parallelepiped cell $P_{0}$ and Voronoi cell $V_{0}$, respectively, let the following hold:*

(8) $$A_{\lambda }=V_{0}\cap P_{\lambda }\;\;and\;\;B_{\lambda }=P_{0}\cap V_{\lambda }\;\;for\;\;\lambda \in \Lambda .$$

*Then,*

(9) $$B_{\lambda }=A_{-\lambda }+\lambda .$$

From Proposition 1, we conclude that $vol\left(P_{0}\right)=vol\left(\cup_{\lambda \in \Lambda }B_{\lambda }\right)=vol\left(\cup_{\lambda \in \Lambda }A_{-\lambda }\right)=vol\left(V_{0}\right)$. In particular, we have the following result.

**Proposition** **2.** *The partitions of the hexagonal lattice* Λ *given by the parallelogram partition and Voronoi partition are equivalents.*

**Figure 2:**
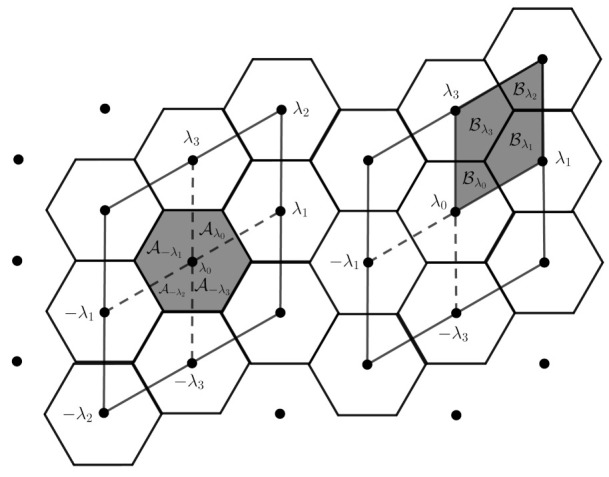
Equivalence between Voronoi and fundamental regions associated to the hexagonal lattice Λ.

**Proof.**  Let $\beta =\{u_{1},u_{2}\}$ be the basis of the hexagonal lattice Λ. We consider $\lambda_{0},\lambda_{1},-\lambda_{1},\lambda_{2},$$-\lambda_{2},\lambda_{3},-\lambda_{3}\in \Lambda_{1}$, where $\lambda_{0}=\left(0,0\right),\lambda_{1}=u_{1},\lambda_{3}=u_{2}$ and $\lambda_{2}=\lambda_{1}+\lambda_{3}$. On the left in the Figure 2, we have four regular hexagons given by the Voronoi cells of the Voronoi partition $\left\{V_{\lambda }:\lambda \in \Lambda \right\}$, where $V_{0}=\left\{x\in R^{2}:Q_{\Lambda_{1}}\left(x\right)=\lambda_{0}\right\},V_{1}=\left\{x\in R^{2}:Q_{\Lambda_{1}}\left(x\right)=\lambda_{1}\right\},V_{2}=\left\{x\in R^{2}:Q_{\Lambda_{1}}\left(x\right)=\lambda_{2}\right\},V_{3}=\left\{x\in R^{2}:Q_{\Lambda_{1}}\left(x\right)=\lambda_{3}\right\}$. On the right, we have four parallelograms given by the fundamental cell of partition $\left\{P_{\lambda }:\lambda \in \Lambda \right\}$, denoted by $P_{\lambda_{0}},P_{-\lambda_{1}},P_{-\lambda_{2}},P_{-\lambda_{3}}$. By $P_{\lambda_{i}}$, we denote the parallelogram whose lower point in the left edge of the parallelogram is the lattice point $\lambda_{i}$.

**Figure 3:**
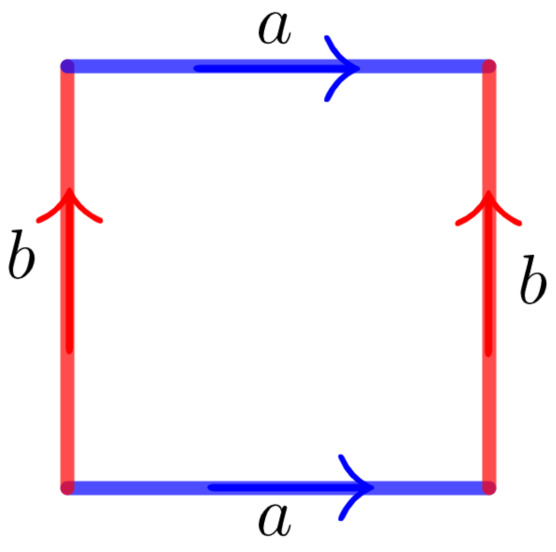
Edge identifications to obtain the 2D-torus.

Let $A_{\lambda }=V_{0}\cap P_{\lambda }$, for $\lambda =\lambda_{0},-\lambda_{1},-\lambda_{2},-\lambda_{3}\in \Lambda_{1}$. Then, $A_{\lambda_{0}}=V_{0}\cap P_{\lambda_{0}}$, $A_{-\lambda_{1}}=V_{0}\cap P_{-\lambda_{1}}$, $A_{-\lambda_{2}}=V_{0}\cap P_{-\lambda_{2}}$, $A_{-\lambda_{3}}=V_{0}\cap P_{-\lambda_{3}}$. Taking $B_{\lambda }=P_{0}\cap V_{\lambda }$ for $\lambda =\lambda_{0},\lambda_{1},\lambda_{2},\lambda_{3}\in \Lambda_{1}$, we have $B_{\lambda_{0}}=V_{0}\cap V_{\lambda_{0}}$, $B_{\lambda_{1}}=P_{0}\cap V_{\lambda_{1}}$, $B_{\lambda_{2}}=P_{0}\cap V_{\lambda_{2}}$, $B_{\lambda_{3}}=P_{0}\cap V_{\lambda_{3}}$. By Proposition 1, $B_{\lambda }=A_{-\lambda }+\lambda$. Thus, for the lattice points $\lambda \in \Lambda_{1}$ given in Figure 3, we have $B_{\lambda_{0}}=A_{-\lambda_{0}}+\lambda_{0}$, $B_{\lambda_{1}}=A_{-\lambda_{1}}-\lambda_{1}$, $B_{\lambda_{2}}=A_{-\lambda_{2}}-\lambda_{2}$, $B_{\lambda_{3}}=A_{-\lambda_{3}}-\lambda_{3}$. This implies that the fundamental hexagon $V_{0}$ is equivalent to the fundamental region $P_{0}$, giving that the partitions are equivalent. □

## 3. Tessellations of the Flat Torus

Here, we give definitions and general results on tessellations in the Euclidean space $R^{n}$, and in the flat torus.

**Definition** **4.** *Let G be a discrete group of isometries acting on a metric space X. A closed set F⊂X with non-empty interior $F^{\circ }$ is a fundamental region for G if it satisfies the following conditions:*

*1.*  *$\cup_{T\in G}T\left(F\right)=X$;*
*2.*  *$F^{\circ }\cap {T(F)}^{\circ }=\emptyset$, for every, T∈G−{Id}. The family {T(F):T∈G} is called a tessellation of X.*

A covering of *X* by copies of *F* under the action of a group of isometries *G* is called a tessellation of *X* associated to *G*, or a *G*-tessellation.

Let Λ be one of the lattices $Z^{2},\;Z^{3}$ or $Z^{4}$. By Remark 3, these lattices Λ induce two different but equivalent tessellations (paralellepiped partitioning P and Voronoi partitioning V) in $R^{2}$, $R^{3}$ and $R^{4}$, respectively. Furthermore, these tessellations are self-dual. In these three cases, the discrete group acting on them is the same (see Costa [17]), that is, G≃Λ, the group of translations generated by these basis, considering the tessellation given by congruent paralellepiped to the paralellepiped $P_{0}$ with support in the basis β, or considering the tessellation given by the Voronoi regions congruent to the Voronoi region $V_{0}$ with center in the lattice point 0 (one vertex of the parallelogram $P_{0}$).

In a similar way, the hexagonal lattices Λ of Example 4 induce two equivalent tessellations in $R^{2}$ by parallelograms (paralellepiped partition P) and regular hexagons (Voronoi partition V) with the same discrete group G≃Λ.

We denote by {p,q} a tessellation in $R^{2}$, where the fundamental region *F* is a regular polygon with *p* sides, and where in each vertex we have the meet of *q* copies of *F*. The {4,4}, {6,3} and {3,6} are all possible regular tessellations {p,q} in $R^{2}$. The tessellation {q,p} is the dual tessellation of {p,q}.

From a geometrical point of view, in the dual tessellation {q,p}, the barycenters of the regular polygons with *p* sides of {p,q} become vertices of the regular polygons with *q* sides, where each vertex is covered by *p* regular polygons of *q* sides. In $R^{2}$, the tessellation {4,4} is dual of itself, and it is called self-dual tessellation.

The region *F* of Definition 4, in each case, can be seen as the closure, in relation to the Euclidean norm, of the fundamental regions of the two partitions considered in Examples 2, 3 and 4.

Voronoi partitions of lattice $Z^{2}$, and the sublattices $\Lambda^{\prime }$ with index $m^{2}$ in $Z^{2}$, described in item (1) of Example 3, are given by squares, whose baricenters are points of the lattice, and reproduce a tessellation {4,4} in $R^{2}$.

On the other hand, the parallelogram partitioning associated both for the lattice $Z^{2}$ and for the sublattices $\Lambda^{\prime }$ of index $m^{2}$ in $Z^{2}$, in item (1) of Example 3, also reproduces a regular tessellation {4,4} in $R^{2}$, where the vertices of the squares are lattice points.

The Voronoi partitioning associated with the hexagonal lattice Λ as well as for the sublattices $\Lambda^{\prime }$ of index $m^{2}$ in Λ, described in item (1) of Example 4, and for the sublattices $\Lambda^{\prime \prime }$ with index of $9m^{2}$ in Λ, described in in item (2) of Example 4, are given by regular hexagons, whose barycenters are lattice points and reproduce a regular tessellation {6,3} in $R^{2}$.

The partitioning by parallelograms associated also with the hexagonal lattice Λ and with the sublattices $\Lambda^{\prime }$ of index $m^{2}$ in Λ, described in item (1) of Example 4, as well as with the sublattices $\Lambda^{\prime \prime }$ with index $9m^{2}$ in Λ, described in item (2) of Example 4, are given by parallelograms, which also reproduce a non-regular tessellation in $R^{2}$, where the vertices of the parallelograms are points of the hexagonal lattice.

The lattices of type Λ obtained from a basis β, described in Examples 1, 3 and 4, induce two different but equivalent tessellations (parallelogram partitioning and Voronoi partitioning) in $R^{2}$. In both cases, the discrete group acting on them is the same (see Costa [17]), that is, $G_{1}≃\Lambda$, the group of translations generated by the basis β, considering the tessellation given by congruent parallelograms to the parallelogram $P_{0}$ with support in the basis β, or considering the tessellation given by the Voronoi regions congruent to the Voronoi region $V_{0}$ with center in the lattice point 0 (one vertex of the parallelogram $P_{0}$). In a similar way, the lattice $\Lambda^{\prime }$ of Example 4 induces two tessellations in $R^{2}$ by parallelograms and regular hexagons with the same discrete group $G≃\Lambda^{\prime }$.

The flat torus, which is a concern to us, is defined on the fundamental region $P_{0}$ of the lattice parallelogram partition, either from $Z^{2}$ or from the hexagonal lattices of basis $\beta^{\prime }$. However, our interest is to obtain regular tessellations in the flat torus from the regular tessellations of $R^{2}$. For this reason, Lemma 1 and Proposition 1 are of fundamental importance.

Using a basis $\gamma =\{u_{1},u_{2},\ldots ,u_{n}\}$ of an *n*-dimensional lattice Λ and a basis $\gamma^{\prime }=\left\{u_{1}^{\prime },\ldots ,u_{n}^{\prime }\right\}$ of a sublattice $\Lambda^{\prime }$ of Λ, the flat torus $T_{\gamma }$ is defined as the quotient space $T_{\gamma }=R^{n}/\Lambda$. For the quotient, we consider the map $\mu :R^{n}\to R^{n}$ defined as $\mu_{\gamma }\left(x\right)=x\;\;mod\;\;\Lambda =x-\sum_{i=1}^{n}\left[x_{i}\right]u_{i}$, where $x=\sum_{i=1}^{n}x_{i}u_{i}$ and $\left[x_{i}\right]$ denotes the greatest integer less than or equal to *x*. Thus, $x,y\in R^{n}$ are in the same coset in $T_{\gamma }$ if, and only if, $\mu_{\gamma }\left(x\right)=\mu_{\gamma }\left(y\right)$, i.e., $x-y=\sum_{i=1}^{n}m_{i}u_{i},\;\;m_{i}\in Z$. The flat torus $T_{\gamma }$ can be characterized as the quotient of the Euclidean space $R^{n}$ by a group of translations.

Now, considering the torus $T_{\gamma }$ generated by the basis γ, we can also define the quotient map $\bar{\mu_{\gamma }}:R^{n}\to T_{\gamma }$ using the fundamental region $P_{0}$ in $R^{n}$, given by the basis γ.

Considering the sublattices $\Lambda^{\prime }$ of basis $\beta^{\prime }$, obtained from lattice Λ with basis β, described in Examples 3 and 4, we can see $T_{\beta }$ as the parallelogram generated by the sides supported by vectors $u_{1},\ldots ,u_{n}$ since we identify the opposite sides.

**Figure 4:**
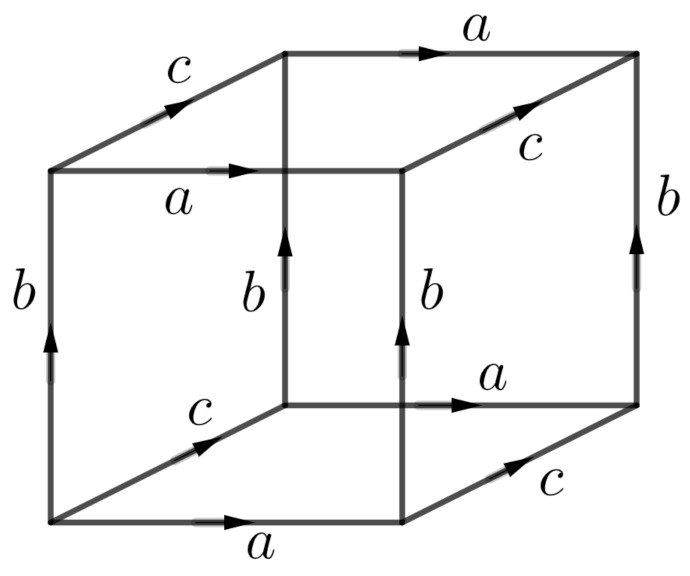
Face identifications to obtain the 3D-torus.

The flat torus (2D torus) in the plane can be constructed from a square by identifying each pair of opposite edges (see Figure 3). The flat torus (3D torus) in the space can be constructed from a cube by identifying each pair of opposite faces (see Figure 4).

Let Λ be a lattice with basis γ and $\Lambda^{\prime }$ a sublattice of Λ with basis $\gamma^{\prime }$. The next result, from Costa et al. [17], shows that, under some conditions, it is possible to obtain tessellations on the flat torus $T_{\gamma }$ induced by the tessellation associated with the lattice $\Lambda^{\prime }$ in $R^{n}$.

**Proposition** **3.** *Let $\gamma =\{u_{1},\ldots ,u_{n}\}$ and $\gamma^{\prime }=\left\{u_{1}^{\prime },\ldots ,u_{n}^{\prime }\right\}$ be bases of the lattices* Λ *and $\Lambda^{\prime }$, respectively. We consider the* Λ*-tessellation of $R^{n}$, which has as a fundamental region the polytope $P_{0}$ supported on γ. If $\Lambda^{\prime }$ is a sublattice of* Λ*, and $\bar{\mu_{\gamma^{\prime }}}$ is the quotient map on the flat torus $T_{\gamma^{\prime }}$, then* Λ *induces a G-tessellation on the flat torus $T_{\gamma^{\prime }}=R^{2}/\Lambda^{\prime }$ with fundamental region $\bar{\mu_{\gamma^{\prime }}}\left(P_{0}\right)$, where $G=\Lambda /\Lambda^{\prime }$.*

Based on Remark 1 and Proposition 3, we will determine, using Proposition 4, families of $Z_{m}^{n}$-tessellations derived from $Z^{n}$-lattices (n=2,3,4). We will also obtain families of $Z_{m}^{2}$-tessellations $T_{\beta^{\prime }}$ on each flat torus $T_{\beta^{\prime }}$, $Z_{3m}^{2}$-tessellations on each flat torus and $T_{\beta^{\prime \prime }}$ derived from hexagonal lattices.

**Proposition** **4.** *Let* Λ *be any lattice of $R^{n}$ with basis $\beta =\{u_{1},u_{2},\ldots ,u_{n}\}$ and let $\Lambda^{\prime }$ be a sublattice of index $m^{n}$(m>1) with $\Lambda^{\prime }$ with basis $\beta^{\prime }=\left\{mu_{1},mu_{2},\ldots ,mu_{n}\right\}$. Then, the lattice *Λ* induces a $Z_{m}^{n}$-tessellation in each flat torus $T_{\beta^{\prime }}≃R^{n}/\Lambda^{\prime }$, where to the lattice quotient, we have $\Lambda /\Lambda^{\prime }≃Z_{m}^{n}$.*

**Proof.**  If $v^{\prime }\in \Lambda$, then $v^{\prime }=a_{1}u_{1}+a_{2}u_{2}+\cdots +a_{n}u_{n}$, for some $a_{1},a_{2},\ldots ,a_{n}\in Z$. For each m≥2, we can write $v^{\prime }=\left(mk_{1}+r_{1}\right)u_{1}+\left(mk_{2}+r_{2}\right)u_{2}+\cdots +\left(mk_{n}+r_{n}\right)u_{n}$, where $a_{i}=mk_{i}+r_{i}$ for some $k_{1},k_{2},\ldots ,k_{n}\in Z$ and $r_{1},r_{2},\ldots ,r_{n}\in \left\{0,\ldots ,m-1\right\}$. Taking the quotient homomorphism $f_{1}$, the following holds:

(10) $$f_{1}:\Lambda ⟶\Lambda /\Lambda^{\prime },$$

where each $v^{\prime }=a_{1}u_{1}+a_{2}u_{2}+\cdots +a_{n}u_{n}\in \Lambda$ can be written in the quotient lattice $\Lambda /\Lambda^{\prime }$ as $w=f\left(v^{\prime }\right)=r_{1}u_{1}+r_{2}u_{2}+\cdots +r_{n}u_{n}$. Finally, let us consider the following homomorphism:

(11) $$f_{2}:\Lambda /\Lambda^{\prime }⟶Z_{m}\times Z_{n}\times \ldots \times Z_{m}\;\left(m\;times\right)$$

which takes $w=r_{1}u_{1}+r_{2}u_{2}+\cdots +r_{n}u_{n}\in \Lambda /\Lambda^{\prime }$ to $w^{\prime }=f_{2}\left(v^{\prime }\right)=\left(r_{1},r_{2},\ldots ,r_{n}\right)\in Z_{m}\times Z_{m}\times \ldots \times Z_{m}$, where $r_{1},r_{2},\ldots ,r_{n}\in \left\{0,1,\ldots ,m-1\right\}$. It is easy to prove that $f_{2}$ is an isomorphism, that is, $\Lambda /\Lambda^{\prime }≃Z_{m}^{n}$. Thus, by Proposition 3, the lattice $\Lambda_{\beta }$ induces a $Z_{m}^{n}$-tessellation in each flat torus $T_{\beta^{\prime }}≃R^{n}/\Lambda^{\prime }$. □

**Corollary** **1.** *By Proposition 4, we have the following:*

*1.*  *The lattice $\Lambda =Z^{2}$ induces a $Z_{m}^{2}$-tessellation in each flat torus $T_{\beta^{\prime }}≃R^{2}/\Lambda^{\prime }$ for the family of sublattices with basis $\beta^{\prime }$ and index $m^{2}$ in the lattices* Λ*, as described in item (1) of Example 3.*
*2.*  *The lattice $\Lambda =Z^{3}$ induces a $Z_{m}^{3}$-tessellation in each flat torus $T_{\beta^{\prime }}≃R^{3}/\Lambda^{\prime }$ for the family of sublattices with basis $\beta^{\prime }$ and index $m^{3}$ in the lattices* Λ*, as described in item (2) of Example 3.*
*3.*  *The lattice $\Lambda =Z^{4}$ induces a $Z_{m}^{4}$-tessellation in each flat torus $T_{\beta^{\prime }}≃R^{4}/\Lambda^{\prime }$ for the family of sublattices with basis $\beta^{\prime }$ and index $m^{4}$ in the lattices* Λ*, as described in item (3) of Example 3.*
*4.*  *The hexagonal lattice* Λ *induces a $Z_{m}^{2}$-tessellation in each flat torus $T_{\beta^{\prime }}≃R^{2}/\Lambda^{\prime }$ to the sublattice family with basis β and index $m^{2}$ in the lattice* Λ*, as described in item (1) of Example 4.*
*5.*  *The hexagonal lattice* Λ *induces a $Z_{m}^{2}$-tessellation in each flat torus $T_{\beta^{\prime }}≃R^{2}/\Lambda^{\prime }$ to the sublattice family with basis β and index $m^{2}$ in the lattice* Λ*, as described in item (1) of Example 4.*
*6.*  *The hexagonal lattice* Λ *induces a $Z_{3m}^{2}$-tessellation in each flat torus $T_{\beta^{\prime }\prime }≃R^{2}/\Lambda^{\prime \prime }$ to the sublattice family with basis β and index $9m^{2}$ in the lattice* Λ*, as described in item (2) of Example 4.*

### 3.1. $Z_{m}^{n}$ Regular Tessellations of the Flat Torus Derived from $Z^{n}$-Lattices

In this subsection, for the cases n=2,3 and n=4, we show that each sublattice $\Lambda^{\prime }$ with basis $\beta^{\prime }$ in $Z^{n}$ determines a flat torus $T_{\beta^{\prime }}≃R^{n}/\Lambda^{\prime }$, using fundamental paralellepiped $P_{0}$ of the paralellepiped partition P, associated with each sublattice $\Lambda^{\prime }=mZ^{n}$. Propositions 5–7 determine the $Z_{m}^{n}$ regular tessellations of the flat torus derived from $Z^{n}$-lattices for the cases n=2,3 and n=4.

We recall that, if $P_{0}$ is a fundamental region in $R^{n}$, then it is identified in each flat torus $T_{\beta^{\prime }}$ in the form $\bar{\mu_{\beta^{\prime }}}\left(P_{0}\right)$, where $\bar{\mu_{\beta^{\prime }}}$ is the quotient application considered from a lattice $\Lambda^{\prime }$, with basis $\beta^{\prime }$, as described in Proposition 4.

**Proposition** **5.** *Let $\Lambda =Z^{2}$ be the lattice with basis $\beta =\{u_{1},u_{2}\}$ and let $\Lambda^{\prime }$ be a sublattice of index $m^{2}$ in* Λ*, with basis $\beta^{\prime }=\left\{mu_{1},mu_{2}\right\}$. Then, the lattice $Z^{2}$ induces a $Z_{m}^{2}$ regular tessellation in the flat torus $T_{\beta^{\prime }}$, given by $m^{2}$ squares.*

**Proof.**  Let $P_{0}$ e $P_{0}^{\prime }$ be the fundamental regions of $Z^{2}$ and $\Lambda^{\prime }$, respectively. From Corollary 1, $Z^{2}$ induces a $Z_{m}^{2}$-tessellation in the flat torus $T_{\beta^{\prime }}$, and from Proposition 4, the quotient lattice $\Lambda /\Lambda^{\prime }≃Z_{m}^{2}$ has carnality $m^{2}$. On the other hand, by Equation (3), $|\Lambda /\Lambda^{\prime }|=\frac{vol(P_{0}^{\prime })}{vol(P_{0})}=m^{2}$. Thus, the fundamental region $P_{0}^{\prime }$ is covered by $m^{2}$ squares, which are congruent to the fundamental region $P_{0}$. □

With similar reasoning, we prove the next two propositions.

**Proposition** **6.** *Let $\Lambda =Z^{3}$ be the lattice with basis $\beta =\{u_{1},u_{2},u_{3}\}$ and let $\Lambda^{\prime }$ be a sublattice of index $m^{3}$ in* Λ*, with basis $\beta^{\prime }=\left\{mu_{1},mu_{2},mu_{3}\right\}$. Then, the lattice $Z^{3}$ induces a $Z_{m}^{3}$ regular tessellation in the flat torus $T_{\beta^{\prime }}$, given by $m^{3}$ cubes.*

**Proposition** **7.** *Let $\Lambda =Z^{4}$ be the lattice with basis $\beta =\{u_{1},u_{2},u_{3},u_{4}\}$ and let $\Lambda^{\prime }$ be a sublattice of index $m^{4}$ in* Λ*, with basis $\beta^{\prime }=\left\{mu_{1},mu_{2},mu_{3},mu_{4}\right\}$. Then, the lattice $Z^{4}$ induces a $Z_{m}^{4}$ regular tessellation in the flat torus $T_{\beta^{\prime }}$, given by $m^{4}$ hypercubes.*

### 3.2. $Z_{m}^{2}$ Regular Tessellations of the Flat Torus Derived from Hexagonal Lattices

In this subsection, we show that the hexagonal sublattices $\Lambda^{\prime }$ with basis $\beta^{\prime }$ in the hexagonal lattice Λ determines a flat torus $T_{\beta^{\prime }}≃R^{2}/\Lambda^{\prime }$, using regular polygons from tessellations {6,3} and {3,6} in $R^{2}$. We also show that the hexagonal sublattice $\Lambda^{\prime \prime }$ with basis $\beta^{\prime \prime }$ in the hexagonal lattice Λ determines a flat torus $T_{\beta^{\prime \prime }}≃R^{2}/\Lambda^{\prime \prime }$, using regular polygons from tessellation {6,3} in $R^{2}$.

We recall that, if $P_{0}$ is a fundamental region in $R^{n}$, then it is identified in each flat torus $T_{\beta^{\prime }}$ in the form $\bar{\mu_{\beta^{\prime }}}\left(P_{0}\right)$, where $\bar{\mu_{\beta^{\prime }}}$ is the quotient application considered from a lattice $\Lambda^{\prime }$, with basis $\beta^{\prime }$, as described in Proposition 4.

**Proposition** **8.** *Let* Λ *be a hexagonal lattice with basis $\beta =\{u_{1},u_{2}\}$, and let $\Lambda^{\prime }$ be a sublattice of index $m^{2}$ in* Λ *, with basis $\beta^{\prime }=\left\{mu_{1},mu_{2}\right\}$. Then, the lattice* Λ *induces a $Z_{m}^{2}$ regular tessellation in the flat torus $T_{\beta^{\prime }}$, given by $m^{2}$ regular hexagons.*

**Proof.**  Let $P_{0}$ and $P_{0}^{\prime }$ be the fundamental regions of Λ and $\Lambda^{\prime }$, respectively. From Corollary 1, Λ induces a $Z_{m}^{2}$ tessellation in the flat torus $T_{\beta^{\prime }}$, and by Proposition 4, the quotient lattice $\Lambda /\Lambda^{\prime }≃Z_{m}^{2}$ has cardinality $m^{2}$. Now, from Equation (3), $|\Lambda /\Lambda^{\prime }|=\frac{vol(P_{0}^{\prime })}{vol(P_{0})}=m^{2}$. Lemma 1 gives that the fundamental region $P_{0}$ and the Voronoi cell $V_{0}$ associated to the hexagonal lattice Λ are congruent. Thus, the respective fundamental regions $P_{0}^{\prime }$ and the Voronoi cells $V_{0}^{\prime }$, associated to each sublattice $\Lambda^{\prime }$ of Λ, are also congruent.

Thus, the fundamental region $P_{0}^{\prime }$ is covered by $m^{2}$ regular hexagons congruent to the Voronoi region $V_{0}$. Therefore, the lattice $Z^{2}$ induces a $Z_{m}^{2}$ regular tessellation in the flat torus $T_{\beta^{\prime }}$, given by $m^{2}$ regular hexagons. □

**Corollary** **2.** *Let* Λ *be a hexagonal lattice with basis $\beta =\{u_{1},u_{2}\}$ and let $\Lambda^{\prime \prime }$ be a sublattice of index $9m^{2}$ in* Λ*, with basis $\beta^{\prime \prime }=\left\{3mu_{1},3mu_{2}\right\}$. Then, the lattice* Λ *induces a $Z_{3m}^{2}$ regular tessellation in the flat torus $T_{\beta^{\prime \prime }}$, given by $9m^{2}$ hexagons.*

Note that the $Z_{3m}^{2}$ regular tessellation in the flat torus $T_{\beta^{\prime \prime }}$ given by Corollary 2 is covered by $9m^{2}$ regular hexagons and it is derived from the regular tessellation {6,3} in $R^{2}$.

**Example** **6.** *Let* Λ *be a hexagonal lattice with basis $\beta =\{u_{1},u_{2}\}$, and let $\Lambda^{\prime \prime }$ be a sublattice of index 9 in* Λ*, with basis $\beta^{\prime \prime }=\left\{3u_{1},3u_{2}\right\}$, where $u_{1}=\left(0,\sqrt{3}\right)$ and $u_{2}=\left(\frac{3}{2},\frac{\sqrt{3}}{2}\right)$. Then, the lattice* Λ *induces a $Z_{3}^{2}$ regular tessellation in the flat torus $T_{\beta^{\prime \prime }}$, given by 9 hexagons.*

In the next proposition, we have a connection with the dual tessellation {3,6}.

**Proposition** **9.** *Let* Λ *be a hexagonal lattice with basis $\beta =\{u_{1},u_{2}\}$, and let $\Lambda^{\prime }$ be a sublattice of index $m^{2}$ in* Λ*, with basis $\beta^{\prime }=\left\{mu_{1},mu_{2}\right\}$. Then, the flat torus $T_{\beta^{\prime }}$ is tessellated by $2m^{2}$ triangles.*

**Proof.**  Let $P_{0}$ and $P_{0}^{\prime }$ be the fundamental regions of Λ and $\Lambda^{\prime }$, respectively. From Proposition 8, the lattice Λ induces a $Z_{m}^{2}$ regular tessellation in the flat torus, given by $m^{2}$ hexagons, that is, the fundamental region $P_{0}^{\prime }$ is covered by $m^{2}$ regular hexagons congruent with the Voronoi region $V_{0}$.

**Figure 5:**
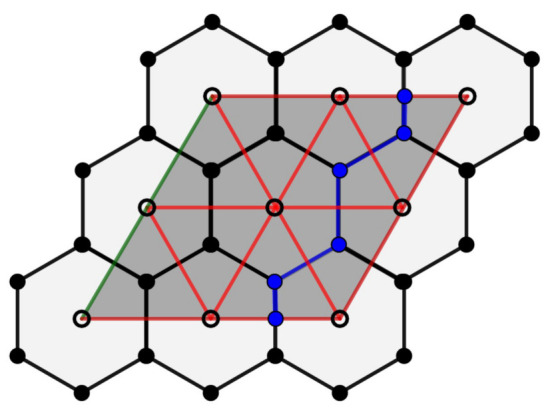
$Z_{2}\times Z_{2}$-tessellation of the flat torus $T_{\beta^{\prime }}$ simultaneously covered by 4 hexagons and 8 triangles.

On other hand, as we can see in Figure 5, for the case m=2, each parallelogram in the quotient space is covered by two triangles. Since the same occurs for m>2, we conclude that the fundamental region $P_{0}^{\prime }$ associated to the sublattice $\Lambda^{\prime }$ of Λ is covered by $2m^{2}$ regular triangles. □

## 4. Toric Codes from the Flat Torus

In this section, for the cases n=2,3 and n=4, we reproduce the toric codes through the flat-torus tessellation $Z_{m}^{n}$, obtained as a consequence of the lattice partition $R^{n}/\Lambda^{\prime }$, where $\Lambda^{\prime }$ is a sublattice of $Z^{n}$ of index $m^{n}$.

### 4.1. Toric Codes from the Flat Torus Derived from $Z^{2}$-Lattices

The toric codes proposed by Kitaev [4] are obtained from $Z_{m}^{2}$-tessellations from flat torus $T_{\beta^{\prime }}$. As given in Proposition 5, the lattice $Z^{2}$ induces a $Z_{m}^{2}$ regular tessellation in the flat torus, that is, the flat torus $T_{\beta^{\prime }}$ is covered by $m^{2}$ squares, and it is derived from the regular tessellation {4,4} of $R^{2}$. From Section 3, we have that the regular tessellation {4,4} comes from the partition of parallelograms obtained from $Z^{2}$, and its associated dual tessellation is also in the form {4,4}, and it comes from the Voronoi partition of the lattice $Z^{2}$.

In the construction of toric codes, the qubits are in biunivocal correspondence with the edges of the $m^{2}$ squares that cover the flat torus $T_{\beta^{\prime }}$. This class of codes has parameters $\left[\left[2m^{2},2,m\right]\right]$, where the length of the code is given by the number of edges of $Z_{m}^{2}$ regular tessellation, that is, $2m^{2}$. The number of information qubits depends on the genus of the oriented surface gT, and since in the flat torus we have g=1, then k=2g=2 qubits are encoded. The distance is given by the minimum between the edges contained in the smallest homologically non-trivial cycle of the $Z_{m}^{2}$ regular tessellation of the flat torus, where we have $2n^{2}$ squares derived from regular tessellation {4,4} in $R^{2}$. Since the tessellation {4,4} is auto-dual, we have that the homologically non-trivial cycle is the path taken by the edges that cannot be contracted on a face. The shortest of these two paths corresponds to the orthogonal axes of the $Z_{m}^{2}$ regular tessellations of the flat torus $T_{\beta^{\prime }}$. Thus, we obtain that d=m.

### 4.2. Toric Codes from the Flat Torus Derived from $Z^{3}$-Lattices

The 3D toric codes were studied in [11]. They are obtained from $Z_{m}^{3}$-tessellations from flat torus $T_{\beta^{\prime }}$. As given in Proposition 6, the lattice $Z^{3}$ induces a $Z_{m}^{3}$ regular tessellation in the flat torus, that is, the flat torus $T_{\beta^{\prime }}$ is covered by $m^{3}$ cubes, and it is derived from the regular tessellation by cubes of $R^{3}$. From Section 3, we have that the regular tessellation by cubes is obtained by the partition of the paralellepiped from $Z^{3}$ or by the Voronoi partition from $Z^{3}$. These tessellations are self-dual.

In the construction of toric codes, the qubits are in biunivocal correspondence with the faces of the $m^{3}$ cubes that cover the flat torus $T_{\beta^{\prime }}$. We can put qubits on faces and checks associated to cubes and edges or we can put qubits on edges and checks on faces and vertices; both choices are related by taking the dual lattice. Thus, regardless of this choice, one logical is like a string (1D torus) and the other is like a sheet (2D torus). The string-like logicals have minimum weight *m* and the sheet-like logicals have minimum weight $m^{2}$. Then, the minimum is *m*, giving the distance. Thus, this class of codes has parameters $\left[\left[3m^{3},3,m\right]\right]$, where the length of the code is given by the number of faces of $Z_{m}^{3}$ regular tessellation, that is, $3m^{3}$ (each face is shared by two cubes).

### 4.3. Toric Codes in the Flat Torus Derived from $Z^{4}$-Lattices

The toric codes proposed in [12] are obtained from $Z_{m}^{4}$-tessellations from flat torus $T_{\beta^{\prime }}$. As given in Proposition 7, the lattice $Z^{4}$ induces a $Z_{m}^{4}$ regular tessellation in the flat torus, that is, the flat torus $T_{\beta^{\prime }}$ is covered by $m^{4}$ hypercubes, and it is derived from the regular tessellation by hypercubes of $R^{4}$. From Section 3, we have that the regular tessellation of hypercubes is obtained by the partition of the paralellepiped from $Z^{4}$ or by the Voronoi partition from $Z^{4}$. These tessellations are self-dual.

Following the reasoning of last subsection, we obtain the toric codes, where the qubits are in biunivocal correspondence with the faces of the $m^{4}$ cubes that cover the flat torus $T_{\beta^{\prime }}$. This class of codes has parameters $\left[\left[6m^{3},6,m^{2}\right]\right]$, where the length of the code is given by the number of faces of $Z_{m^{4}}^{4}$ regular tessellation, that is, $6m^{4}$.

## 5. Toric Codes Derived from Hexagonal Lattices

We have seen that the parallelepiped P and the Voronoi partitions V are associated with the lattices $Z^{n}$ (n=2,3 and n=4) reproduce self-dual tessellations in $R^{n}$. The construction of toric codes from tessellations from the flat torus $Z^{n}$ as well as from its dual tessellation are equivalent.

Now, we propose constructions of the toric and color codes from hexagonal lattices such that they also come from the flat torus tessellations $Z_{m}^{n}$. Although the parallelepiped P and the Voronoi V partitions associated with the hexagonal lattices are not self-dual, they reproduce equivalent tessellations in $R^{n}$, which help us to propose these new codes.

### Toric Codes from $Z_{m}^{2}$-Tessellations of the Flat Torus Derived from Hexagonal Lattices

In this subsection, we extend the toric code construction procedure from the two-dimensional lattices $Z^{2}$ proposed by Kitaev [4] to the hexagonal lattices. Similar to the Kitaev [4] case, we have the qubits in biunivocal correspondence with the edges of the regular polygons that cover the flat torus $T_{\beta^{\prime }}$, where $\beta^{\prime }$ is the basis of the lattice associated to the hexagonal sublattice $\Lambda^{\prime }$ of the hexagonal lattice Λ of index $m^{2}$.

**Proposition** **10.** *Let M be the set of all families covering the flat torus $T_{\beta^{\prime }}$ with $m^{2}$ regular hexagons from the {6,3} tessellation, described by Proposition 8, together with the coverings of the flat torus $T_{\beta^{\prime }}$ with $2m^{2}$ regular triangles from the dual tessellation {3,6}, described by Proposition 9. Then, there are $3m^{2}$ qubits attached at each edge of these regular polygons.*

**Proof.**  We associate the qubits at the edges of the hexagons covering the flat torus $T_{\beta^{\prime }}$ obtained from tessellation {6,3}. Since each hexagon has six edges and each edge is shared between two hexagons, the number of qubits is given by the relation $\frac{6m^{2}}{2}=3m^{2}$.

Similarly, we associate the qubits on the edges of the regular triangles covering the flat torus $T_{\beta^{\prime }}$ obtained from tessellation {3,6}; the number of qubits is given by the relation $\frac{3.2m^{2}}{2}=3m^{2}$. □

We also know that the homological group associated to the flat torus is isomorphic to the group $Z_{2}\times Z_{2}$. By the elementary results of Group Theory, we conclude that the homology group has two generators. Therefore, we obtain that each code C, constructed from each flat torus $T_{\beta^{\prime }}$, encodes k=2 qubits since there are two stabilizer operators in each hexagonal face.

Based on Proposition 10, we obtain in Proposition 11 an algebraic procedure to obtain toric codes from families of the flat torus $T_{\beta^{\prime }}$.

**Proposition** **11.** *From each flat torus $T_{\beta^{\prime }}=R^{2}/\Lambda^{\prime }$, we obtain a toric code with parameters $\left[\left[3m^{2},2,m\right]\right]$.*

**Proof.**  From Proposition 10, we obtain the parameters of the code on each flat torus $T_{\beta^{\prime }}$. Then, we need only calculate the distance of the code.

By definition, the minimum distance of a stabilizer code is the weight of the Pauli operator with minimum weight, which preserves the code subspace and acts non-trivially on it. Since we are working with a special family of homological codes, we can see this distance in function of the homology of the surface; thus, the minimum distance is the least number of qubits in support of a homologically non-trivial cycle between the tessellation and dual tessellation associated on flat torus $T_{\beta^{\prime }}$.

In relation to the covering of the flat torus $T_{\beta^{\prime }}$, obtained from the regular tessellation {3,6}, we have that the homologically non-trivial cycle is the path given by the edges that cannot be contracted into a face. The smallest of these paths corresponds to the axes parallel to the support vectors of the fundamental region of $\Lambda^{\prime }$ which, due to its identification, determines the flat torus $T_{\beta^{\prime }}$. Then, d=m.

We analyze the covering of the flat torus $T_{\beta^{\prime }}$. We prove by induction on *m* that for all $Z_{m}^{2}$-tessellation of the flat torus $T_{\beta^{\prime }}$, the distance is d=2m+1. From Figure 5, we see that d=3 for m=1. Let us suppose by the induction hypothesis that d=2(m−1)+1=2m−1 for m−1.

Now, we prove for *m*. Geometrically, the $Z_{m-1}^{2}$-tessellation can be seen as a subset of $Z_{m}^{2}$-tessellation. Note that, to calculate the distance in the $Z_{m}^{2}$-tessellation, we can start from $Z_{t}^{2}$-tessellation; then, the distance is given by the least number of quibts in support of a non-trivial cycle, and by the induction hypothesis, the distance is given by 2t−1. The remainder of the homologically non-trivial cycle to be traversed in $Z_{m}^{2}$-tessellation is equivalent to the path in the cycle that we have already seen for the case m=1, where we have that d=3. Then, the distance is 2m−1+3=2m+2 but, in the construction from m−1 to *m*, we have counted an edge twice, so we instead obtain d=2m+1. Therefore, the minimum distance is d=m. □

**Figure 6:**
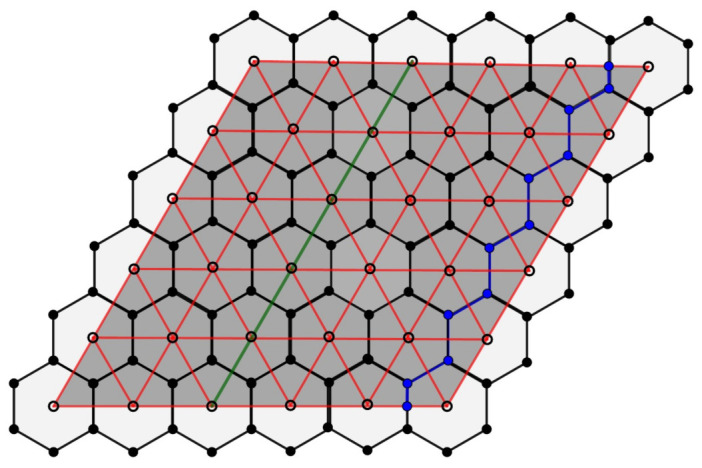
Color code from $T_{\beta^{\prime }}$.

**Remark** **4.** *The green paths in Figure 5 and Figure 6 describe the non-trivial cycles with a minimum length in the flat torus para $\beta^{\prime }=\left\{2u_{1},2u_{2}\right\}$ e $\left\{5u_{1},5u_{2}\right\}$, respectively, given by regular triangles from the regular {3,6} of $R^{2}$. In a similar way, in the blue paths, we consider the regular hexagons from the regular tessellation {6,3}. In Figure 6, we obtain distance 5 in the green cycle, considering the covering by 50 triangles, and we obtain distance 11 in the blue cycle, considering the covering by 25 hexagons.*

## 6. Color Codes from $Z_{3m}^{2}$-Tessellation of the Flat Torus Derived from Hexagonal Lattices

In the construction of color codes from the flat torus, we need a three-valent tessellation, which has three-colorable faces. We have that the $Z_{3m}^{2}$-tessellation of the flat torus $T_{\beta^{\prime \prime }}$ has these properties. Thus, in this section, we give an algebraic/geometric procedure to build quantum color codes, with parameters $\left[\left[18m^{2},4,4m\right]\right]$ from tessellations $Z_{3m}^{2}$ on the flat torus $T_{\beta^{\prime \prime }}$.

Figure 2 shows a tessellation by regular hexagons (fundamental cell of the lattice), where the barycenters of the hexagons are the points of the lattice. On the left we see that, from a regular hexagon, through convenient rearrangements, we obtain a parallelogram, which is a fundamental cell of the lattice. On the other hand, in the right in the figure, we have that the smallest parallelogram is a fundamental cell of the lattice, such that the parallelogram in the larger region is the fundamental cell of the sublattice. The index of the lattice by the sublattice gives the amount of parallelograms that covers the larger one (equivalently, the number of hexagons in the larger parallelogram). Each of the smallest parallelograms contains two vertices of a tessellation by hexagons, where the quibts are indexed, as we can see in the right of the figure. The next proposition gives a control on the faces in the $Z_{3m}^{2}$-tessellations.

**Proposition** **12.** *Let M be the set given by the coverings of the flat torus $T_{\beta^{\prime \prime }}$, where each covering has $9m^{2}$ regular hexagons from the {6,3} tessellation as described by Corollary 2. Then, we have $18m^{2}$ qubits attached in the edges of these regular polygons.*

**Proof.**  By Corollary 2, when considering the vectors of the basis $\beta^{\prime \prime }$ of the family of sublattices $\Lambda^{\prime \prime }$ of the hexagonal lattices Λ parallel to the vectors of basis $\beta^{\prime \prime }$, and since the length of the vectors in $\beta^{\prime \prime }$ is three times the length of the vectors of basis β, we have that the $Z_{3m}^{2}$-tessellation is three-colorable. The topology of the quotient group $\Lambda /\Lambda^{\prime \prime }$ leads to $9m^{2}$ coset representatives on each flat torus $T_{\beta_{\prime \prime }}$. Consequently, we conclude there are two qubits placed on each coset representative. Therefore, there are $18m^{2}$ qubits placed on the each flat torus $T_{\beta_{\prime }}$. □

We also know that the homological group associated to the flat torus is isomorphic to the group $Z_{2}\times Z_{2}$. By the elementary results of Group Theory, we conclude that the homology group has two generators. Therefore, we obtain that each code C, constructed from each flat torus $T_{\beta^{\prime \prime }}$, encodes k=4 qubits since there are two stabilizer operators in each hexagonal face.

Based on Proposition 12 and Corollary 2, we obtain in Proposition 13 an algebraic procedure to obtain color codes from families of the flat torus $T_{\beta^{\prime \prime }}=R^{2}/\Lambda^{\prime \prime }$.

**Proposition** **13.** *From each flat torus $T_{\beta^{\prime \prime }}=R^{2}/\Lambda^{\prime \prime }$, we obtain a color code with parameters $\left[\left[18m^{2},4,4m\right]\right]$.*

**Proof.**  From Corollary 2, we obtain the parameters of the code on each flat torus $T_{\beta^{\prime \prime }}$, where $\beta^{\prime \prime }=\left\{mu_{1},mu_{2}\right\}$ is the lattice basis associated to $\Lambda^{\prime \prime }$. Then, we need only calculate the distance of the code, as we did in Proposition 11. Considering the fundamental region $P_{0}^{\prime }$ tiled by $Z_{3m}^{2}$-tessellations, induced by the Λ-tessellation, for the calculation of the minimum distance, we make use of mathematical induction.

We consider as an induction hypothesis that the minimum distance of a code obtained from a flat torus $T_{\beta^{\prime \prime }}$ is given by d=4(m−1) for the case (m−1), i.e., $\beta^{\prime \prime }=\left\{\left(m-1\right)3u_{1},\left(m-1\right)3u_{2}\right\}$. When m=1, we have $\beta^{\prime \prime }=\left\{3u_{1},3u_{2}\right\}$. The fundamental region $P_{0}$ associated with the lattice is given by the parallelogram $P_{0}$.

**Figure 7:**
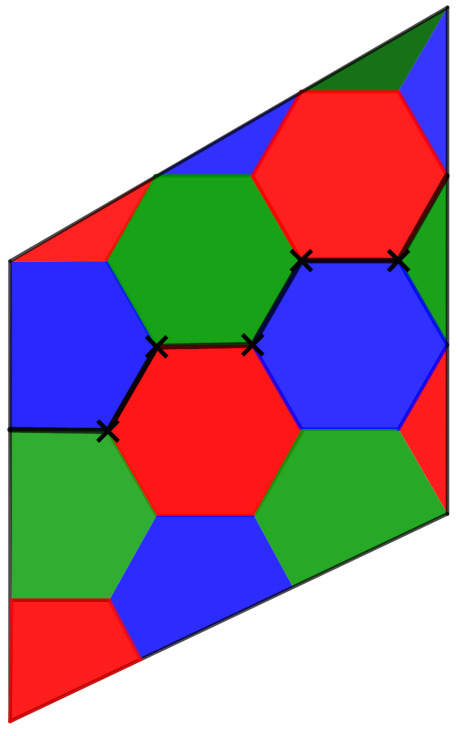
Color code with distance 4 from $Z_{3}^{2}$-tessellation of flat torus $T_{\beta^{\prime \prime }}$.

As can be seen in Figure 7, the minimum distance is d=⌊4.1⌋=4. Then, the code C obtained from the case m=1 has parameters [[18,4,4]]. The cycle traverses the hexagonal vertices that contain the vector $u_{1}$ of basis $\beta_{1}$. Analogously, we could consider the cycle through the hexagonal vertices containing the vector $u_{2}$ of basis $\beta_{3}$.

For the case m−1, let us first recall that the Λ induces in the flat torus $T_{\beta^{\prime \prime }}$ a $Z_{3(m-1)}^{2}$-tessellation, and in the flat torus. Geometrically, the $Z_{3(m-1)}^{2}$-tessellation can be seen as a subset of $Z_{3m}^{2}$-tessellation. To calculate the distance in the $Z_{3m}^{2}$-tessellation, we can start from $Z_{3(n-1)}^{2}$-tessellation; then, the distance is given by the least number of quibts in support of a non-trivial cycle, and by the induction hypothesis, the distance is given by 4(m−1). The remainder of the homologically non-trivial cycle to be traversed in $Z_{3m}^{2}$-tessellation is equivalent to the path in the cycle that we have already seen for the case m=1, where we have that d=4.

Thus, in the flat torus $T_{\beta^{\prime \prime }}$, we obtain a code with parameters $\left[\left[18m^{2},4,4m\right]\right]$. □

**Example** **7.** *To the case m=1, Figure 7 shows the minimum distance d=4 of the code defined on the flat torus $T_{\beta^{\prime \prime }}$, obtained in Example 6.*

## 7. Conclusions and Discussion

In this work, we proposed an algebraic and geometric characterization of the construction of topological quantum codes from a family of flat tori since we may characterize topological codes on the flat torus as cosets of lattice quotients, putting them in the context of lattice theory. These facts have not yet been exploited in quantum coding theory. The lattice theory is a rich algebraic and geometric theory. Among the geometric properties, we were interested in lattice partitions, with emphasis on the parallelepiped and Voronoi partitions—different and equivalent ways of covering the space $R^{n}$. We obtained several families of toric codes in dimensions 2,3 and 4 and color codes on hexagonal lattices.

## Data Availability

Not applicable.
